# Prognostic and clinicopathological significance of long noncoding RNA H19 overexpression in human solid tumors: evidence from a meta-analysis

**DOI:** 10.18632/oncotarget.13076

**Published:** 2016-11-04

**Authors:** Fang-teng Liu, Hua Pan, Guang-feng Xia, Cheng Qiu, Zheng-ming Zhu

**Affiliations:** ^1^ Department of General Surgery, the Second Affiliated Hospital of Nanchang University, Nanchang 330000, Jiangxi Province, P. R. China

**Keywords:** long noncoding RNA, H19, carcinoma, prognosis, meta-analysis

## Abstract

**Background:**

Many studies have reported that the expression level of lncRNA H19 was increased in various tumors. LncRNA H19 may play a significant role in cancer occurrence and development. An increased level of H19 was also associated with poor clinical outcomes of cancer patients.

**Results:**

12 eligible studies were screened, with a total of 1437 cancer patients. From the results of meta-analysis, as for prognosis, the patients with high expression of lncRNA H19 were shorter in OS (HR=1.08, 95% CI: 1.05-1.12). Statistical significance was also showed in subgroup meta-analysis stratified by the cancer type, analysis type and sample size. In addition, the patients detected with high H19 expression may be poorer in DFS (HR=1.27; 95% CI = 0.97-1.56). As for clinicopathology, it showed that increased H19 was related to poor histological grades (OR=2.31, 95% CI: 1.12-4.75), positive lymph node metastasis (OR=2.29, 95 % CI: 1.21-4.34) and advanced clinical stage (OR=4.83, 95% CI: 3.16-7.39).

**Materials and Methods:**

Eligible studies were collected by retrieving keywords in PubMed, Web of Science, Embase, CNKI and Wanfang database, from 1966 to April 23, 2016. This quantitative meta-analysis was performed with Stata SE12.0 and RevMan5.3 software. It aimed to explore the association between H19 expression level and prognosis and clinicopathology.

**Conclusions:**

LncRNA-H19 may be a novel molecular marker for predicting solid tumors. It can also be a predictive factor of clinicopathological features in various cancers. Further studies are needed to verify the clinical utility of H19 in human cancers.

## INTRODUCTION

With the development of sequencing technique, it revealed that a majority of human genome was actively transcribed into noncoding RNAs (ncRNAs). Long noncoding RNAs (lncRNAs) was one type of ncRNAs, which attracted widespread interest and attention in current days [1, 2]. The lncRNAs was more than 200 nucleotides in length without protein-coding capacity [3]. Many studies showed that lncRNAs play vital roles in diverse biological processes, as well as in disease mechanism [4–7]. Especially in cancers, some lncRNAs were proved to act as tumor promoter or suppressor in the progress of tumorigenesis [8, 9]. It reported that dys-regulated expression of lncRNAs was involved in apoptosis, proliferation and invasion of cancer cells [10–12]. LncRNA could be served as promising biomarkers for diagnosis and potential therapeutic targets in cancers. However, biological functions of most lncRNAs remained far from clear.

H19, also known as BWS, ASM1 and ASM, is a paternally imprinted gene, which locates in 11p15.5 [13]. The long noncoding RNA H19 was encoded by this highly conserved imprinted gene. In recent years, increasing studies have reported that lncRNA-H19 was up-regulated in various cancers, such as non-small cell lung cancer, bladder cancer, breast cancer and gastric cancer [14–17]. The tumor cell proliferation, invasion and metastasis would be promoted with up-regulated expression of lncRNA-H19. It suggested that lncRNA-H19 may be functioned as an oncogene in tumorigenicity [18–20]. The expression level of lncRNA-H19 in cancerous tissues was determined to be significantly higher than that of in adjacent normal tissues. In addition, lncRNA-H19 expression was positive associated with poor clinical outcomes in multiple tumors. Therefore, lncRNA-H19 might be feasible as prognostic prediction biomarker.

A meta-analysis has been performed to explore the correlation between lncRNA-H19 expression and clinical outcome in cancer patients. It further discussed whether lncRNA-H19 could be served as a practical biomarker on prognosis prediction in human solid tumors.

## RESULTS

### Main information of included studies

**Figure 1:**
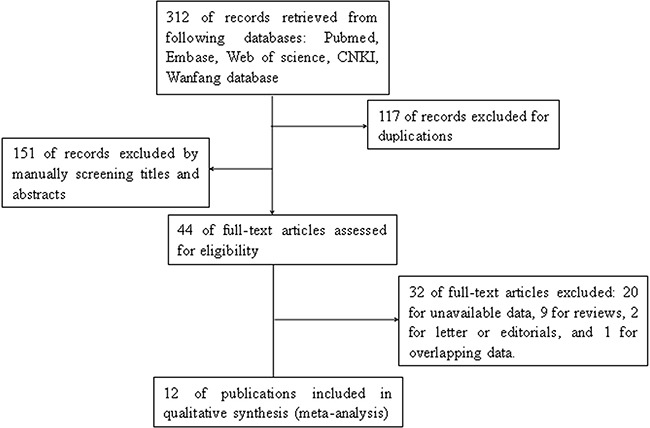
Flowchart indicated the process of literature retrieval and selection

12 studies [21–32] were finally identified according to the inclusion and exclusion criteria. The process of literature retrieval was shown (Figure 1). A total of 1437 patients in 12 studies were included in this analysis. The mean sample size of patients was 119.7 (ranged from 24 to 361). The cancer cases in 12 included studies were all Asians (11 from China, 1 from Korea). In the 12 studies, 9 types of cancer were evaluated: 4 gastric cancer (GC), 1 non-small cell lung cancer (NSCLC), 1 renal cell carcinoma (RCC), 1 ovarian cancer (OC), 1 gallbladder cancer (GBC), 1 laryngeal squamous cell cancer (LSCC), 1 colorectal cancer (CRC), 1 esophageal cancer (EC) and 1 hepatocellular carcinoma (HCC). All these cancer types were solid cancer. The main information and data were summarized (Table 1).

**Table 1:** The main information of included studies in the meta-analysis

Author (Publication year)	Country	Cancer type	Total sample size	Therapy before	Tumor stage (I/II/III/IV)	Follow-up (months)	Outcome measures	Analysis type	Cut-off value of H19 expression	Detection method of H19 expression
Zhang, 2014 [21]	China	GC	80	None	15/21/33/11	Over 60	OS	Multivariate	above the mean	qRT-PCR
Li, 2014 [22]	China	GC	74	N/A	57/17(I–II/III–IV)	1-50	OS	Kaplan-Meier curves	≥6-fold	qRT-PCR
Wang, 2015 [23]	China	RCC	92	N/A	56/36(G1-2/G3-4)	1-60	OS	Multivariate	≥3.8-fold	qRT-PCR
Zhang, 2015 [24]	China	NSCLC	70	N/A	18/32/18/2	1-60	OS	Multivariate	above median	qRT-PCR
Zhu, 2015 [25]	China	OC	70	None	21/49(I–II/III–IV)	N/A	N/A	N/A	N/A	qRT-PCR
Huang, 2015 [31]	China	EC	133	N/A	63/70(0-I/II-IV)	N/A	N/A	N/A	>median	qRT-PCR
Yang, 2015 [30]	Korea	HCC	240	None	102/100/33/5	Over 60	OS.DFS	Univariate (OS); Multivariate (DFS)	≥ median	N/A
Wang, 2016 [26]	China	GBC	24	None	12/12(I–II/III–IV)	1-40	OS	Kaplan-Meier curves	≥ median ratio	qRT-PCR
Chen, 2016 [28]	China	GC	128	N/A	27/53/45/3	median 36	OS,DFS	Multivariate	≥4.47-fold	qRT-PCR
Wu, 2016 [27]	China	LSCC	82	N/A	45/37(I–II/III–IV)	1-60	OS	Kaplan-Meier curves	N/A	qRT-PCR
Li, 2016 [32]	China	GC	361	N/A	133/228 (G1-2/G3-x)	1-60	OS	Kaplan-Meier curves	N/A	N/A
Han, 2016 [29]	China	CRC	83	N/A	38/45(I–II/III–IV)	Over 40	OS,DFS	Multivariate	≥3-fold	qRT-PCR

**Abbreviations:** GC: gastric cancer; RCC: renal cell carcinoma; NSCLC: non-small cell lung cancer; OC: ovarian cancer; EC: esophageal cancer; HCC: hepatocellular carcinoma; GBC: gallbladder cancer; LSCC: laryngeal squamous cell cancer; CRC: colorectal cancer; OS: overall survival; Multivariate: multivariate analysis; Univariate: univariate analysis; N/A::not available

### The determination of H19 in included studies

The comparison of H19 expression level from included studies was important in meta-analysis. In these studies, known expression level of H19 was determined with qRT-PCR. The high expression of H19 in individual study was provided (Table 1). It slightly differed in different papers. However, the results from these studies could be still comparable. The determination of H19 expression was influenced by following factors: 1. Determination principle, for the included studies, the determination principle was the same, which was qRT-PCR. 2. Primer and potential probes, the primer and probes may be slightly different. However, the highly conserved sequence of H19 enabled similar amplification efficiency, even with slightly varied primer and probe sequence. 3. Determination rules, such as cutoff value, most of the cutoff value was determined as median value of adjacent control tissues.

### Association between lncRNA-H19 expression and OS

**Figure 2:**
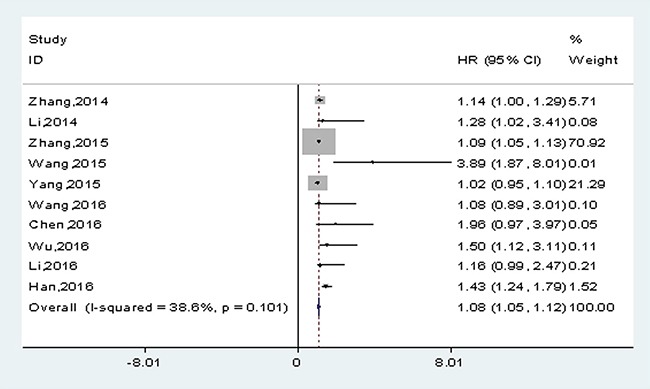
Meta-analysis of the pooled HRs of OS in solid cancers

In 12 included studies, 10 studies reported the prognosis of OS according to H19 expression levels, with a total of 1234 patients. There was no significant heterogeneity among studies (*I^2^*=38.6%, *P_h_*=0.101). The fixed-effects model was adopted to calculate the pooled HRs with corresponding 95% confidence intervals (CIs). The HRs was expressed as high H19 expression group versus low H19 expression group, which was 1.08 (95% CI: 1.05-1.12, *p*< 0.001) (Figure 2).

**Figure 3:**
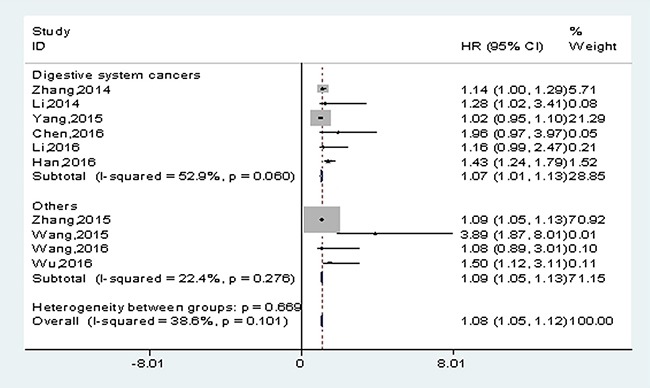
Meta-analysis for the pooled HRs of OS in patients with various cancers

A subgroup analysis was performed based on the types of cancer. A positive result was observed in patients with gastric cancer (HR: 1.15, 95% CI: 1.01-1.28, *p*<0.001) (Table 2, the figure was presented in Supplement information Figure S1). When all cancer types were generally classified into 2 categories (digestive system cancers and others), a similar result was found in digestive system cancers (HR: 1.07, 95% CI: 1.01-1.13, *p*<0.001) (Figure 3). It suggested that the up-regulated lncRNA-H19 expression was positively correlated with poor prognosis in patients with digestive system cancers.

**Table 2:** Meta-analysis results of the associations of lncRNA-H19 with OS according to subgroup analysis

Categories	Studies (n)	Number of patients	Fixed-effects model		Heterogeneity	
			HR (95% CI) for OS	*p*-value	*I^2^* (%)	*P_h_*
[1] OS	10	1234	1.08(1.05-1.12)	< 0.001	38.6	0.101
[2] Cancer type						
1) Digestive system cancers	6	966	1.07(1.01-1.13)	< 0.001	52.9	0.060
Others	4	268	1.09(1.05-13)	< 0.001	22.4	0.276
2) Gastric cancer	4	643	1.15(1.01-1.28)	< 0.001	0.0	0.756
Others	6	591	1.08(1.04-1.11)	< 0.001	60.3	0.027
[3] Analysis type						
Multivariate	5	453	1.10(1.06-1.14)	< 0.001	62.9	0.029
Non-multivariate	5	781	1.03(0.96-1.10)	< 0.001	0.0	0.883
[4] Sample size						
≥ 100	3	729	1.03(0.96-1.10)	< 0.001	0.0	0.449
< 100	7	505	1.10(1.06-1.14)	< 0.001	41.5	0.115

Moreover, for OS, the pooled HRs was significantly and consistently higher than 1 in subgroup meta-analysis, which was stratified by the analysis type and sample size (Table 2, the two figures were presented in Supplementary information Figure S2, S3).

**Figure 4:**
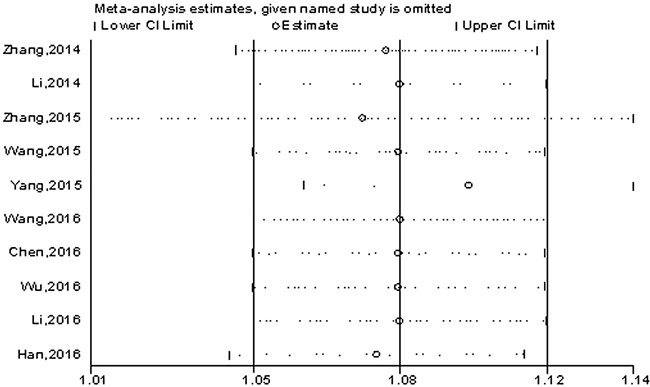
Sensitivity analysis on the relationship between lncRNA-H19 expression and OS

From the overall results, a significant difference of OS was observed between the two groups. OS was prone to reduced in the patients with high H19 expression than that of with low H19 expression. Higher H19 expression was positively correlated with worse survival in various cancers. Sensitivity analysis for OS in solid tumors showed no significant changes in HRs when any individual study was excluded (Figure 4).

### Association between lncRNA-H19 expression and DFS

**Figure 5:**
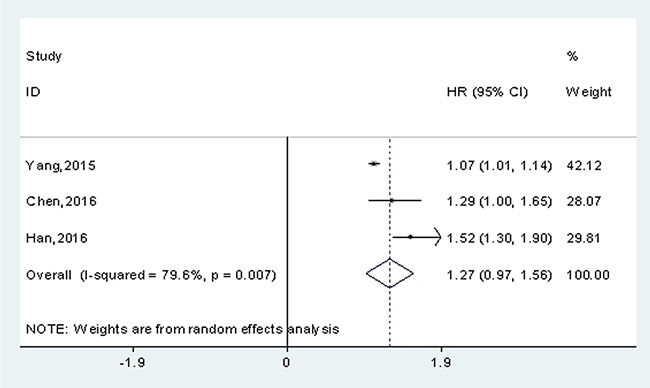
Meta-analysis for the pooled HRs of DFS

In this meta-analysis, 3 studies reported the data of HR values and corresponding 95 % CIs, with a total of 451 patients. Therefore, they were directly applied for evaluating the association between lncRNA-H19 expression and DFS. Because there was significant statistical heterogeneity across-studies (*I^2^*=79.6%; *P_h_* =0.007), the random-effects model was applied. The pooled HR revealed that there may be a significantly positive association between high expression level of lncRNA-H19 and poor DFS (HR=1.27, 95% CI = 0.97-1.56, *p* < 0.001) (Figure 5).

### Association between lncRNA-H19 expression and clinicopathological parameters

The pooled ORs and 95% CIs of clinicopathological parameters in human solid tumors were showed (Table 3). From meta-analysis results, it found that the over-expressed H19 was associated with poor histological grade (OR=2.31, 95% CI: 1.12-4.75, *p*=0.02), positive lymph node metastasis (OR=2.29, 95% CI: 1.21-4.34, *p*=0.01), and advanced TNM stage (OR=4.83, 95% CI: 3.16-7.39, *p*<0.001). However, no significant correlation was observed between H19 expression and gender or distant metastasis (*p*>0.05) (All those figures were presented in Supplement information Supplementary Figure S4–S8). The correlation of H19 expression and other clinicopathological parameters could not be obtained for insufficient information from current studies.

**Table 3:** Meta-analysis results for the associations of over-expressed lncRNA-H19 with clinicopathological parameters

Clinicopathological parameters	Studies (n)	Number of patients	OR (95% CI)	*p*-value	Heterogeneity		
					***I^2^* (%)**	***P_h_***	**Model**
Gender (male vs. female)	7	610	0.90(0.65-1.25)	0.54	0	3.6	Fixed effects
Tumor differentiation (poorly/others vs. well/moderately)	6	467	2.31(1.12-4.75)	0.02	62	13.24	Random effects
Lymph node metastasis (+ vs.-)	7	547	2.29(1.21-4.34)	0.01	62	15.68	Random effects
Distant metastasis (+ vs.-)	4	436	0.83(0.20-3.46)	0.80	81	15.6	Random effects
TNM stage (III-IV vs. I-II)	6	455	4.83(3.16-7.39)	<0.001	0	3.56	Fixed effects

### Publication bias

**Figure 6:**
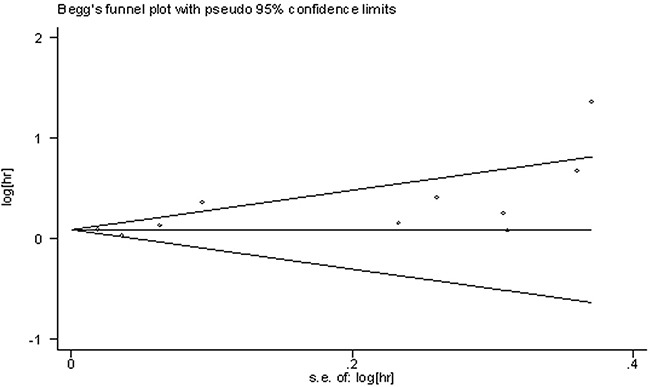
Funnel plots of publication bias on the correlation between lncRNA-H19 expression and OS

The publication bias for OS in solid cancers was evaluated with funnel plots, Begg's and Egger's tests. The Begg's funnel plots revealed no publication bias among these studies (z =1.70, Pr > |z| = 0.089, Figure 6), but the p-value of Egger's test for OS showed a slight publication bias among included studies (*p*=0.039). Then, the trim and fill method was further applied to test for publication bias. The results showed no significant publication bias across studies. Because the number of included studies was limited (n<10), the publication bias for clinicopathological parameters was not assessed.

## DISCUSSION

LncRNA-H19 has been identified as one cancer-related lncRNAs. The high expression level of H19 was considered to be correlated with diverse human disorders and cancers. Growing evidences showed that this H19 gene-encoded 2.3 kb lncRNA functioned in tumorigenesis and cancer progression. The development of cancer may be promoted by the up-regulated expression of H19 [33–35].

The high expression of lncRNA-H19 may be a biomarker for cancer prognosis. Numerous studies have shown that expression levels of lncRNA-H19 in tumor tissues was significant higher than that of in healthy or para-carcinoma tissues [25, 26, 36, 37]. And the increased expression of H19 in cancer tissues was frequently positively correlated with advanced clinical stage and inversely correlated with patient's prognosis [30, 31, 38].

The H19 was a potential target for exploration of cancer drugs. The migration, proliferation and invasion of the tumor cells were able to be significantly inhibited by the knockdown of H19 in vitro. Whereas, enhanced expression of H19 could promote cell proliferation, invasion and metastasis [39, 40]. Therefore, growing evidences suggested that high-expression of lncRNA-H19 promoted development and progression of certain cancers. It was characterized as one kind of crucial oncogenic lncRNAs. However, the underlying molecular mechanisms on the role of H19 in cancer progression and metastasis remained unclear.

Many studies have tried to explore the function mechanism of H19 on tumorgenesis and development. It suggested that H19 promoted pancreatic cancer metastasis by depressing let-7's suppression on its target HMGA2-mediated EMT [41]. Furthermore, a recent study indicated that upregulated H19 could accelerate bladder cancer metastasis by associating EZH2 and inhibiting expression of -E-cadherin, which was the EMT-induced marker [42]. Chen *et al*. also showed that GC metastasis was promoted by up-regulated H19 *in vitro* partly by regulating the E-cadherin expression [28]. And in osteosarcoma, Li *et al.* revealed that H19 could function as a ceRNA in promoting metastasis by impairing miR-200s activity [43].

H19 was the primary precursor of miR-675 and the biological role of H19 in both humans and mice was also elucidated [44]. This finding was confirmed in human colon cancer cells, and furthermore, H19/miR-675 was demonstrated to inhibit suppressor RB in colorectal carcinogenesis [45]. Another study also showed that GC metastasis could be enhanced by H19 *in vitro* and *in vivo* through H19/miR-675 signalling [22]. In breast cancer, Vennin *et al*. found that H19, through its mediator-miR-675, could promote tumor growth and metastasis by down-regulating c-Cbl and Cbl-b [46]. The H19 was recently reported as a molecular sponge to antagonize let-7 [47], which was well-known as a tumor-suppressive microRNA.

There were still many limitations in this meta-analysis. Firstly, the roles and biological functions of lncRNA-H19 differed in different types of cancer. The heterogeneity may be increased, thus influencing firmness of the results. Secondly, all the studies were retrospective studies, with a relatively small sample size. Thirdly, patients included in this meta-analysis were all Asians, for this reason, the results we obtained may just be representative of Asians. Additionally, potential selection and publication bias may be resulted from diversification of data forms and the preference of positive results in publications. Finally, the cut-off definition for high expression in cancerous tissue was not always consistent.

The present meta-analysis explored that high expression of lncRNA-H19 was positively correlated to poor prognosis and clinicalpathological features. Higher lncRNA-H19 expression was significantly correlated with shorter OS and potential poorer DFS in human solid tumors. Furthermore, the clinicopathological significance of over-expressed lncRNA-H19 was also confirmed in this meta-analysis. Increased expression of lncRNA-H19 was shown to be positively correlated with poor differentiation and advanced clinical stage. The patients determined with higher expression of lncRNA-H19 in cancer tissues might also have higher occurrence probability of LNM. Those results suggested that lncRNA-H19 may be served as a biomarker for both prognosis and clinical pathology. Further studies are still needed to verify the clinical utility of H19 in human cancers.

## MATERIALS AND METHODS

### Literature retrieval strategy

Potentially eligible studies were obtained by retrieving key words in multiple databases. The studies were published from 1966 to April 23, 2016. The databases included PubMed, Web of Science, Embase, CNKI and Wanfang database. Following key words were searched in combinations: long non-codingRNA H19, H19, lncRNA H19, H19 fetal liver mRNA; cancer, tumor, neoplasm; prognostic, outcomes, survival, clinicopathological. Other relevant studies were also obtained by manually screening the references list.

### Inclusion and exclusion criteria

Inclusion criteria: (1) the lncRNA-H19 expression was evaluated in human cancer tissues; (2) patients were divided into high expression group and low expression group according to the expression levels of H19; (3) associations of H19 expression with overall survival (OS), disease-free survival (DFS) or clinicopathological features were described.

Exclusion criteria: (1) duplicate publications; (2) studies without available data; (3) overlapping data; (4) reviews, letters, case reports and expert opinions.

### Date extraction and quality assessment

The data were independently extracted from included studies by two investigators (PH and QC), and any disagreements were discussed and judged by a third investigator (XGF). For each study, the following information and data were recorded: the family name of first author, year of publication, country, ethnicity, cancer type, number of patients, follow-up times, outcome measures, cut-off value of H19 expression, analysis method and clinicopathological parameters (such as tender, tumor differentiation), lymph node metastasis, distant metastasis and TNM stage.

For studies provided the results of OS or DFS, multivariate analysis was considered to be prior to univariate analysis. If only Kaplan-Meier curve was provided, the survival data was extracted with Engauge Digitizer version 4.1. The Newcastle-Ottawa Scale (NOS) was involved for quality assessment. The NOS score was ranged from 0 to 9, and the study with an NOS score ≥ 6 was considered to be of high quality. The quality of all studies included in this meta-analysis ranged from 4 to 9, with a mean value of 6.2.

### Statistical methods

Statistical analysis of hazard ratios (HRs) for OS or DFS, and odd ratios (ORs) for clinicopathological parameters were calculated with Stata SE12.0 and RevMan5.3 software, respectively.

The heterogeneity across-studies were determined by Chisquare-based *Q* test and *I^2^* statistics. A *P* value for *Q* test less than 0.05 indicated significant heterogeneity. The *I^2^* value greater than 50 % was considered as severe heterogeneity, and then the random-effects model was applied. For insignificant heterogeneity among studies (*P_h_*>0.05 or *I^2^*<50%), the fixed effects model was applied. *p*-value less than 0.05 was considered to be statistically significant.

## SUPPLEMENTARY FIGURES AND TABLES


