# Supplementary material for: Prognostic and clinicopathological significance of long noncoding RNA H19 overexpression in human solid tumors: evidence from a meta-analysis
Source: Oncotarget. 2016 Nov 4;7(50):83177–86. doi: 10.18632/oncotarget.13076 (PMC5347760; doi:10.18632/oncotarget.13076)
Supplement: Supplementary file 1 [file oncotarget-07-83177-s001.pdf]

Prognostic and clinicopathological significance of long noncoding RNA H19 overexpression in human solid tumors: evidence from a meta-analysis

SUPPLEMENTARY FIGURES AND TABLES

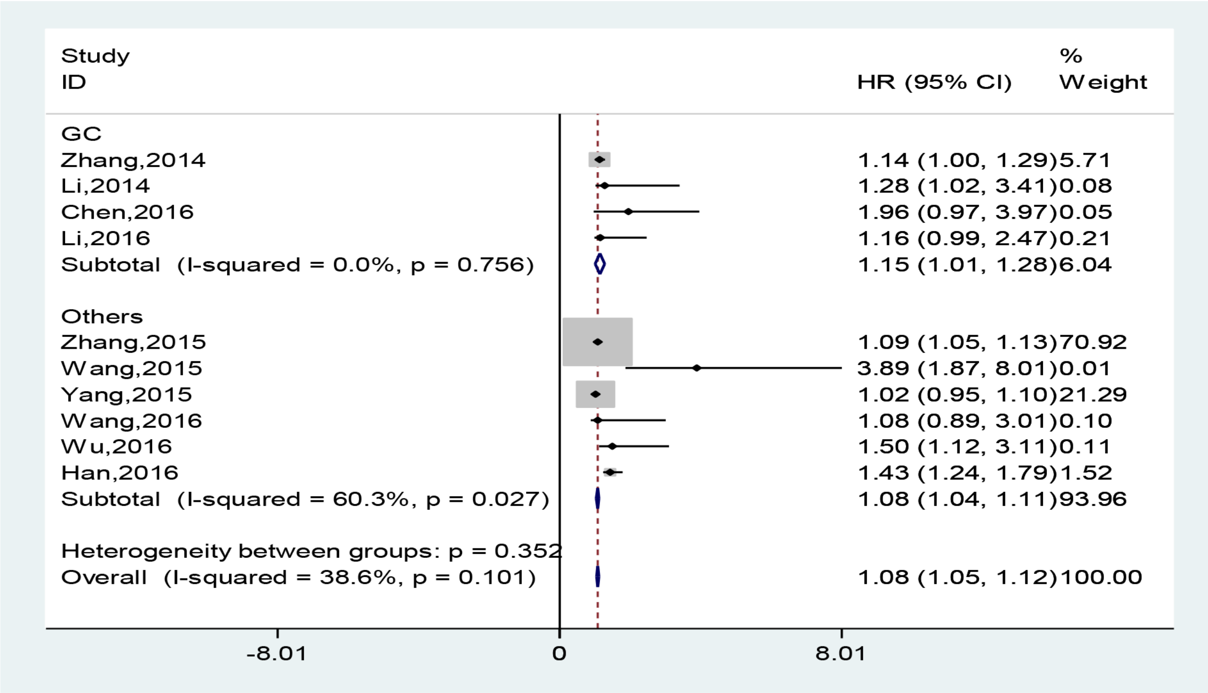

Supplementary Figure S1: Meta-analysis for the pooled HRs of OS stratified by the cancer type.

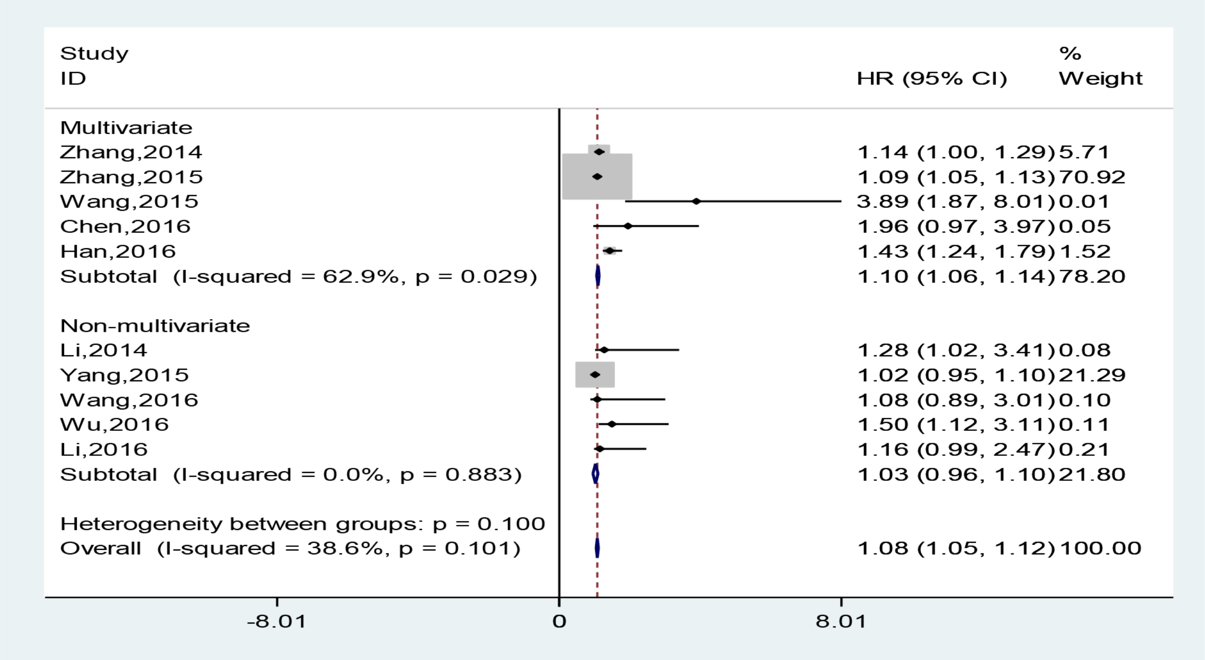

Supplementary Figure S2: Meta-analysis for the pooled HRs of OS stratified by the analysis type.

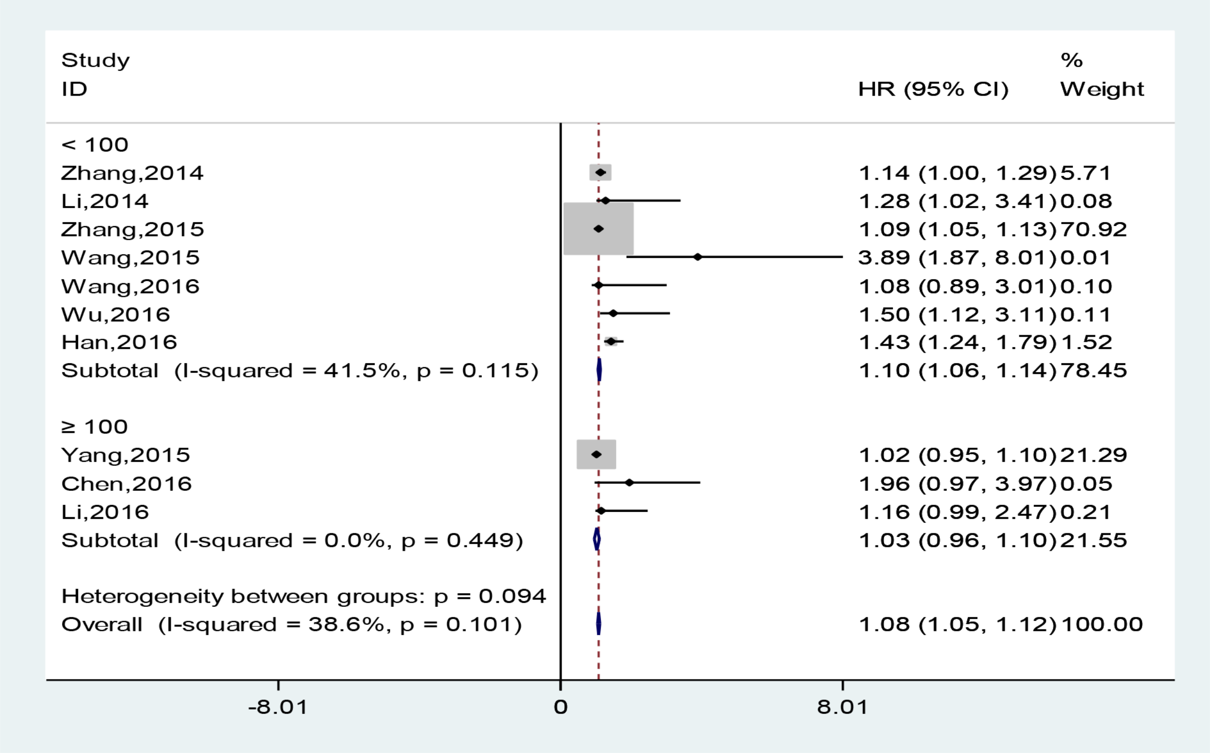

Supplementary Figure S3: Meta-analysis for the pooled HRs of OS stratified by the sample size.

(2) Association between lncRNA-H19 expression and clinicopathological parameters:

Study or Subgroup	Male Events	Male Total	Female Events	Female Total	Weight	Odds Ratio M-H, Fixed, 95% CI	Year
Wang,2015	26	57	16	35	14.1%	1.00 [0.43, 2.32]	2015
Huang,2015	31	65	35	68	23.4%	0.86 [0.44, 1.70]	2015
Zhang,2015	21	46	14	24	13.1%	0.60 [0.22, 1.63]	2015
Zhang,2014	24	47	16	33	12.0%	1.11 [0.46, 2.70]	2015
Chen,2016	41	79	23	49	17.8%	1.22 [0.60, 2.49]	2016
Wang,2016	4	6	9	18	2.0%	2.00 [0.29, 13.81]	2016
Han,2016	20	40	28	43	17.6%	0.54 [0.22, 1.29]	2016
**Total (95% CI)**		**340**		**270**	**100.0%**	**0.90 [0.65, 1.25]**	
Total events	167		141				

Heterogeneity: Chi² = 3.60, df = 6 (P = 0.73); I² = 0%

Test for overall effect: Z = 0.61 (P = 0.54)

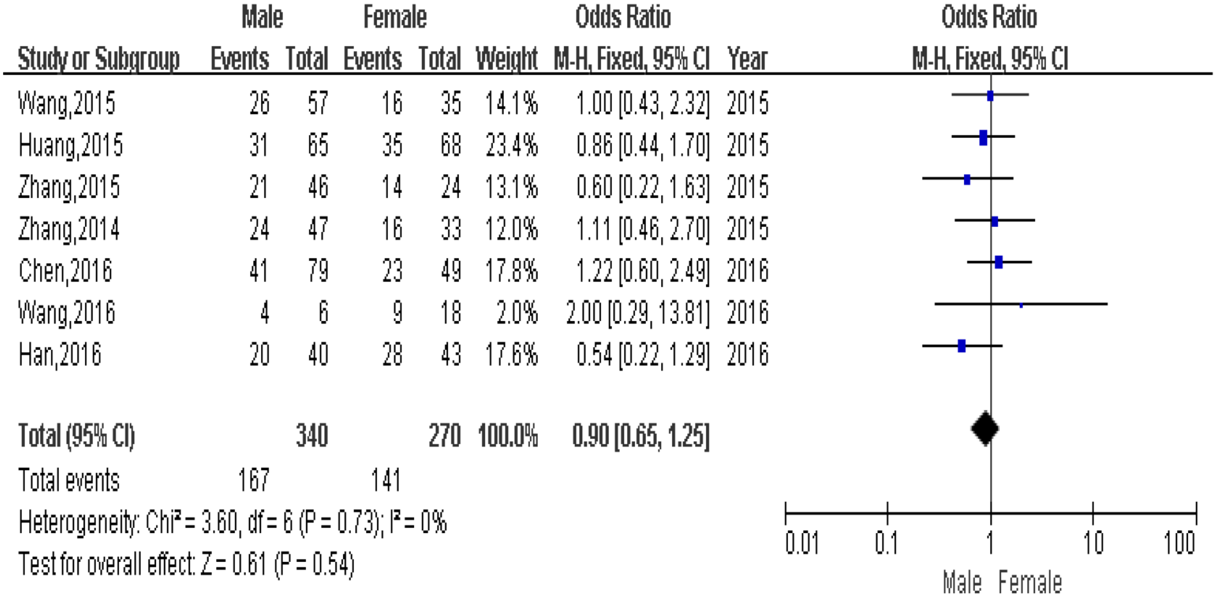

Supplementary Figure S4: The correlation between H19 expression and gender (male vs. female).

Study or Subgroup	Poorly and others Events	Poorly and others Total	Well and morderately Events	Well and morderately Total	Weight	Odds Ratio M-H, Random, 95% CI	Year
Zhu,2015	26	31	31	39	15.5%	1.34 [0.39, 4.60]	2015
Zhang,2015	3	9	32	61	13.0%	0.45 [0.10, 1.98]	2015
Wang,2015	27	36	15	56	18.8%	8.20 [3.14, 21.39]	2015
Wang,2016	9	14	4	10	11.2%	2.70 [0.51, 14.37]	2016
Chen,2016	37	64	27	64	22.3%	1.88 [0.93, 3.79]	2016
Han,2016	36	52	12	31	19.2%	3.56 [1.40, 9.05]	2016
**Total (95% CI)**		**206**		**261**	**100.0%**	**2.31 [1.12, 4.75]**	
Total events	138		121				

Heterogeneity: Tau² = 0.48; Chi² = 13.24, df = 5 (P = 0.02); I² = 62%

Test for overall effect: Z = 2.27 (P = 0.02)

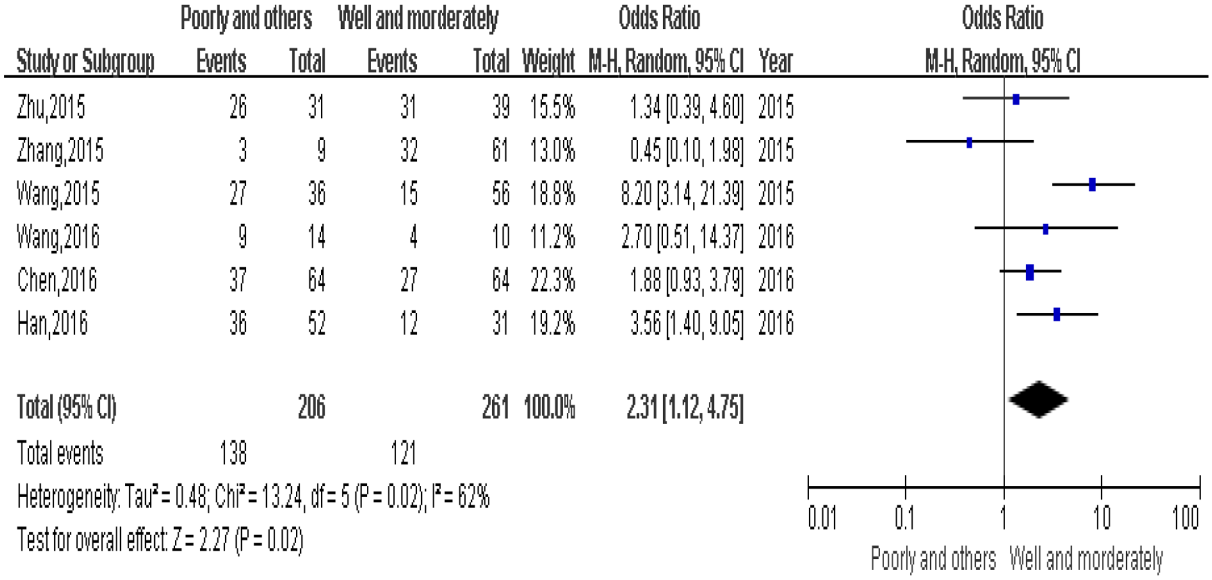

Supplementary Figure S5: The correlation between H19 expression and tumor differentiation (poorly/others vs. well/moderately).

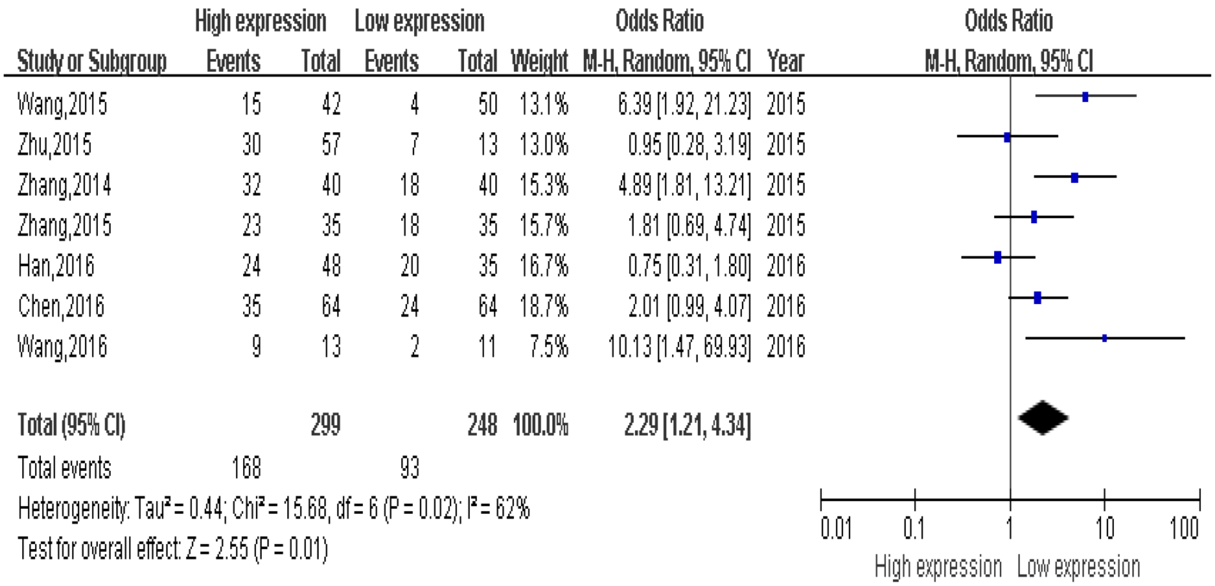

Supplementary Figure S6: The correlation between H19 expression and lymph node metastasis (+ vs.-).

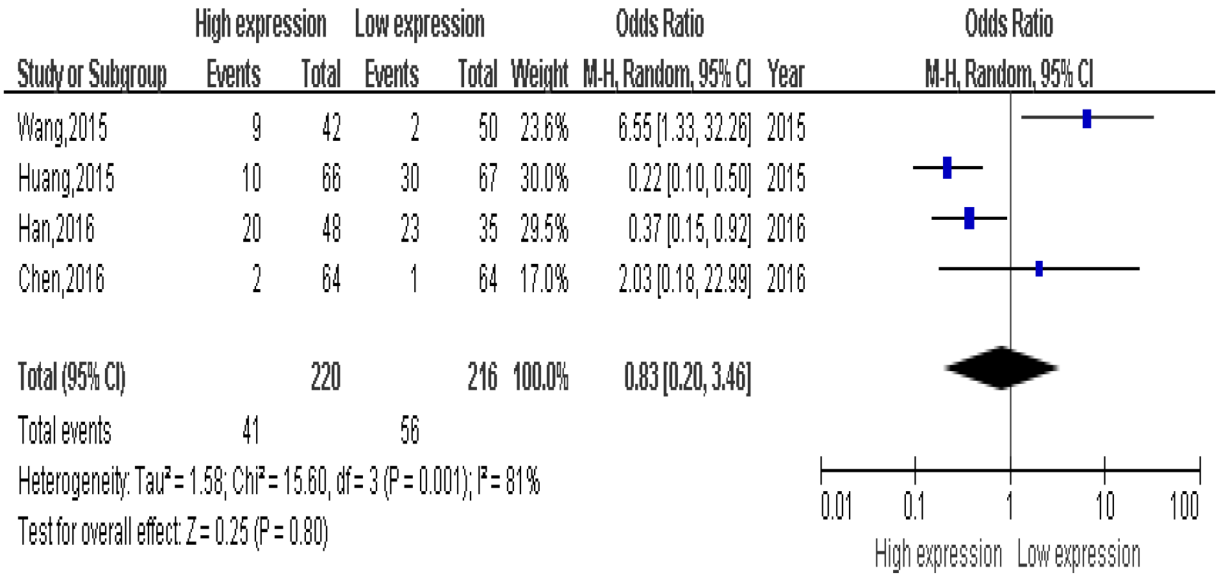

Supplementary Figure S7: The correlation between H19 expression and distant metastasis (+ vs.-).

	III/IV		I/II			Odds Ratio	
Study or Subgroup	Events	Total	Events	Total	Weight	M-H, Fixed, 95% CI	Year
Zhang,2014	31	44	9	36	14.9%	7.15 [2.65, 19.33]	2015
Zhang,2015	17	20	18	50	7.9%	10.07 [2.59, 39.11]	2015
Zhu,2015	44	49	13	21	9.5%	5.42 [1.51, 19.42]	2015
Chen,2016	34	48	30	80	33.4%	4.05 [1.87, 8.74]	2016
Han,2016	31	45	17	38	29.2%	2.74 [1.11, 6.72]	2016
Wang,2016	9	12	4	12	5.1%	6.00 [1.02, 35.37]	2016
**Total (95% CI)**		**218**		**237**	**100.0%**	**4.83 [3.16, 7.39]**	
Total events	166		91				

Heterogeneity: Chi² = 3.56, df = 5 (P = 0.61); I² = 0%

Test for overall effect: Z = 7.26 (P < 0.00001)

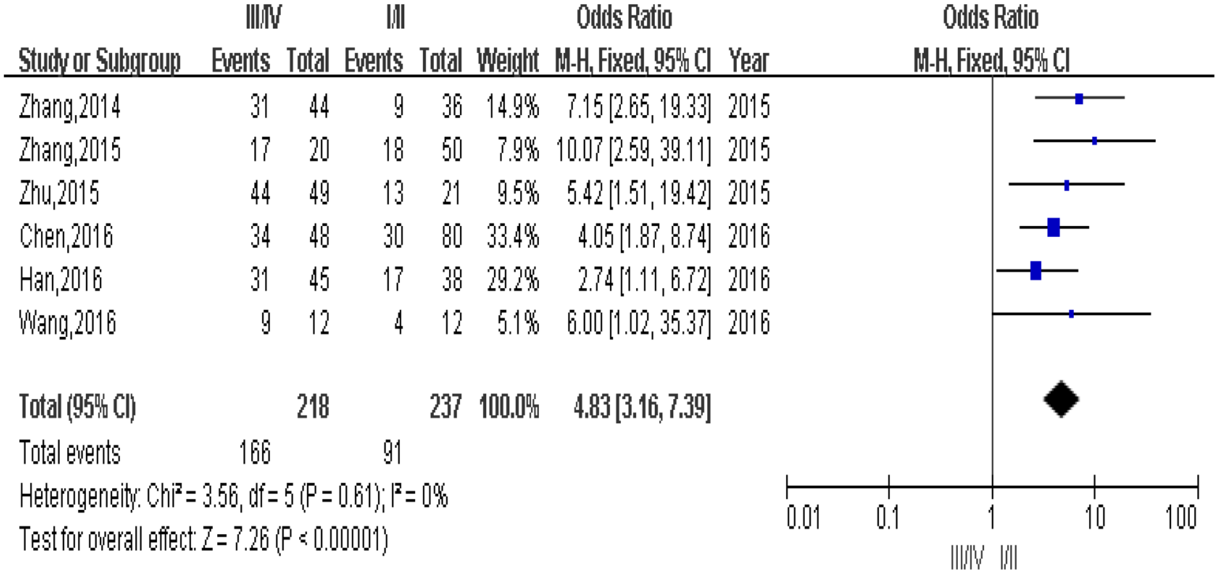

Supplementary Figure S8: The correlation between H19 expression and TNM stage (III-IV vs. I-II).
